# Role of Lipocalin-2 in Brain Injury After Subarachnoid Hemorrhage in Female Mice

**DOI:** 10.3390/cells14221770

**Published:** 2025-11-12

**Authors:** Hao Zhao, Yingfeng Wan, Sravanthi Koduri, Ya Hua, Guohua Xi, Richard F. Keep

**Affiliations:** Department of Neurosurgery, University of Michigan, Ann Arbor, MI 48109, USA

**Keywords:** subarachnoid hemorrhage, lipocalin-2, brain injury, blood–brain barrier

## Abstract

Subarachnoid hemorrhage (SAH) is a devastating cerebrovascular disorder with high mortality and long-term disability. It is more prevalent in women than men, but most preclinical research has been performed in male animals. Upregulation of lipocalin-2 (Lcn2), an acute-phase protein involved in iron homeostasis and neuroinflammation, has been implicated in hemorrhagic brain injury in male animals. The purpose of this study was to examine whether genetic deletion of Lcn2 also reduces early brain injury after SAH in female mice. Adult female wild-type (WT) and Lcn2 knockout (KO) mice were subjected to endovascular perforation to induce SAH. Lcn2 expression was assessed by immunohistochemistry and Western blotting, while brain injury was evaluated using MRI T2 lesion measurement, blood–brain barrier (BBB) permeability assays, Fluoro-Jade C staining, and Garcia’s neurological scoring. We found that Lcn2 expression was upregulated in multiple brain regions after SAH, particularly in astrocytes. Compared with WT mice, Lcn2 KO mice exhibited significantly reduced oxidative stress, attenuated ferritin induction, smaller T2 lesions, decreased BBB leakage, reduced neuronal degeneration, and improved neurological recovery over 7 days. These findings identify Lcn2 as a critical mediator of early brain injury after SAH in female mice. These results further support targeting Lcn2 as a therapeutic strategy to reduce brain damage and improve outcomes in SAH patients.

## 1. Introduction

Subarachnoid hemorrhage (SAH) is a devastating cerebrovascular disease associated with high mortality and long-term disability [1,2]. It is typically caused by rupture of an intracranial aneurysm, leading to rapid increases in intracranial pressure, reduced cerebral perfusion, and secondary brain injury. Clinically, SAH has a higher incidence in women than in men, particularly at older ages, and female patients often experience worse neurological outcomes [2,3]. One study [2] reported a sex-specific incidence rate of SAH of 11.5 per 100,000 person-years (95% CI, 9.5–13.9) in women versus 9.3 per 100,000 person-years (95% CI, 7.7–11.3) in men, with SAH being more prevalent in women than in men in Europe and Asia. Hormonal influences, such as fluctuations in estrogen levels, may affect vascular integrity [4], inflammatory responses, and recovery after hemorrhage, making female subjects an important population for translational relevance. One study measured plasma estrone and estradiol levels in patients with aneurysmal SAH and found that higher estrogen levels correlated with more severe injury and worse outcome [5].

Early brain injury after SAH involves oxidative stress, disruption of the blood–brain barrier (BBB), brain edema, and neuronal cell death, all of which contribute to poor neurological outcomes [6,7,8]. Despite advances in neurosurgical and critical care management, there are still no effective therapies that specifically target the molecular mechanisms underlying early brain injury after SAH. A better understanding of these mechanisms is therefore essential for developing new therapeutic strategies.

One potential target for reducing hemorrhagic brain injury is lipocalin-2 (Lcn2), also known as neutrophil gelatinase-associated lipocalin (NGAL). It is an acute-phase protein that is upregulated in response to infection, inflammation, traumatic brain injury, and intracranial hemorrhage (ICH) [9,10,11]. Increasing evidence suggests that Lcn2 plays an important role in iron homeostasis by regulating iron transport and cellular iron delivery depending on iron availability [12]. Our previous studies [13,14] demonstrated that Lcn2 expression is markedly upregulated after ICH, and that Lcn2 deficiency attenuates early brain injury induced by ICH or thrombin exposure. These findings implicate Lcn2 as a mediator of iron-related toxicity and oxidative stress in hemorrhagic brain injury. However, such studies have focused on male animals and whether Lcn2 contributes to early brain injury following SAH in females has not been well established. Interestingly, a previous clinical study [15] found that cerebrospinal fluid (CSF) Lcn2 levels increased equally in men and women after SAH.

The current study was designed to examine the effects of SAH on Lcn2 expression in brain in female mice and to assess its role in mediating oxidative stress, BBB disruption, neuronal death, and neurological deficits using Lcn2 deficient female mice. We hypothesized that Lcn2 deficiency would attenuate brain injury and improve functional recovery after SAH.

## 2. Materials and Methods

### 2.1. Animals and Subarachnoid Hemorrhage Induction

All animal procedures were approved by the University of Michigan Committee on the Use and Care of Animals. A total of 42 female C57BL/6 mice (Charles River Laboratories, Portage, MI, USA) and 21 female Lcn2 knockout (KO) mice (University of Michigan Breeding Core, generously provided by Dr. Xiaoli Chen, University of Minnesota, Minneapolis, MN, USA), aged 10–12 weeks, weighing 22–30 g, were used. Mice were housed under a 12 h light/dark cycle with free access to food and water. To minimize hormonal variability, all experiments were performed during the diestrus phase. In our study, the individual mouse was treated as the experimental unit for all analyses. Each animal was randomly assigned to a treatment group, and no more than one mouse per litter was used to avoid potential litter-based confounding effects. Mice were obtained from multiple litters and housed in standard groups of 3–5 animals per cage. SAH was induced by a single surgeon using the endovascular perforation method, as previously described [16]. Mice were first anesthetized with 5% isoflurane (VetOne Fluriso; MWI, Boise, ID, USA) and then maintained under continuous isoflurane via a face mask. Core body temperature was maintained at 37 ± 1 °C using a controlled heating pad. With the animals in the supine position, the right external carotid artery (ECA), internal carotid artery (ICA), and common carotid artery (CCA) were exposed. The distal ECA was transected and aligned caudally with the ICA. A 4-0 nylon monofilament suture was introduced into the ECA stump, advanced through the CCA bifurcation into the ICA, and pushed distally until resistance was encountered. The intracranial bifurcation of the ICA was then carefully perforated to induce SAH. The suture was promptly withdrawn into the ECA, which was subsequently coagulated, allowing reperfusion of the ICA. Carprofen (Zoetis Inc., Parsippany, NJ, USA, 5 mg/kg, s.c.) was administered for postoperative analgesia. Following surgery, mice were housed individually for recovery from anesthesia. Sham animals underwent the same procedure without perforation. Mortality was approximately 18% in WT (wild type) and 21% in Lcn2 knockout groups. SAH severity was assessed 24 h post-induction using SAH grading scale [17]. All assessments were performed by blinded investigators.

### 2.2. Immunohistochemistry and Immunofluorescence Staining

The female mice brains were sampled, embedded and sliced into 18 μm thick coronal sections (WT +SAH, *n* = 3; Lcn2-/- +SAH, *n* = 3; Sham, *n* = 3). For immunohistochemistry, primary antibodies were goat anti-Lcn2 antibody (R&D system, Minneapolis, MN, USA, AF1857, 1:200 dilution, RRID:AB_355022). For immunofluorescence double staining, sections were blocked with 10% normal donkey serum for 1 h at room temperature. Primary antibodies were: rabbit anti-Iba-1 (Wako, Richmond, VA, USA, 019-19741, 1:400 dilution, RRID:AB_839504), mouse anti-GFAP (Sigma, Saint Louis, MO, USA, MAB360, 1:400 dilution, RRID:AB_11212597), mouse anti-NeuN (Sigma, Saint Louis, MO, USA, MAB377, 1:400 dilution, RRID:AB_2298772). The secondary antibodies were Alexa Fluor 488-conjugated donkey anti-rabbit mAb (Invitrogen, Carlsbad, CA, USA, A-21206, 1:500 dilution), Alexa Fluor 594-conjugated donkey anti-mouse mAb (Invitrogen, Carlsbad, CA, USA, A-21203, 1:500 dilution). The double-labeling was analyzed using a fluorescence microscope (Olympus BX51 microscope, Evident Scienfitic, Waltham, MA, USA, DP73 digital camera, 40× objectives). Excitation 480 nm and emission 525 nm for green fluorescence, excitation 594 nm and emission 167 nm for red fluorescence. Colocalization was assessed using single optical planes (not z-stacks) to avoid false-positive overlap.

### 2.3. Western Blotting

Western blot analysis was performed as previously described [13]. Briefly, mice were perfused with 0.1 M phosphate-buffered saline (Sigma, Saint Louis, MO, USA, pH 7.4) after euthanasia. Different parts of the brain tissue (white matter and hippocampus) were sampled. Protein concentration was determined by Bio-Rad protein assay kit (Hercules, CA, USA), and 50 μg protein samples were separated by sodium dodecyl sulfate-polyacrylamide gel electrophoresis and transferred onto a hybond-C pure nitrocellulose membrane (Amersham, Pittsburgh, PA, USA). Blocking buffer is 5% non-fat dry milk (Lab Scientific bioKEMIX Inc., Danvers, MA, USA) in PBST. Membranes were probed with the following primary antibodies: goat anti-Lcn2 antibody (R&D system, Minneapolis, MN, USA, AF1857, 1:200 dilution, RRID:AB_355022), rabbit polyclonal anti-HO-1 antibody (Enzo, Farmingdale, NY, USA, ADI-SPA-895-J, 1:4000 dilution, RRID:AB_11180392), rabbit anti-FTH (Ferritin heavy chain) antibody (H-chain, Cell Signaling Technology, Beverly, MA, USA, 4393, 1:2000 dilution, RRID:AB_11217441), polyclonal rabbit anti-FTL (Ferritin light chain) antibody (L-chain, Abnova, Walnut, CA, USA, H00002512-D01P, 1:1000 dilution, RRID:AB_1573649), polyclonal goat anti-mouse albumin antibody (Bethyl Laboratories, Inc., Montgomery, TX, USA, A90-134A 1:10,000 dilution, RRID:AB_2891982), Rabbit anti-rat DARPP32 antibody (Cell Signaling Technology, Danvers, MA, USA, #2306, 1:4000 dilution, RRID:AB_823479), monoclonal mouse anti-beta-actin antibody (Sigma, Saint Louis, MO, USA, A2228 1:250,000 dilution, RRID:AB_476697). Membranes were incubated with primary antibodies overnight at 4 °C, followed by three washes in PBST, each lasting 10 min. The secondary antibodies were goat anti-rabbit IgG (Bio-Rad, Hercules, CA, USA, #172-1019, 1:2000 dilution), rabbit anti-goat (Bio-Rad, Hercules, CA, USA, #1721-043, 1:2000 dilution) and goat anti-mouse IgG (Bio-Rad, Hercules, CA, USA, #170-6515, 1:2000 dilution). Membranes were incubated with secondary antibodies for 1 h at room temperature, followed by three washes in PBST, each lasting 10 min. Antigen–antibody complexes were visualized with the ECL chemiluminescence system (Amersham) and exposed to Kodak X-OMAT film. Exposure times were optimized by serial exposure times to ensure proportional signal intensity. The relative densities of bands were analyzed with ImageJ (National Institutes of Health, Bethesda, MD, USA, version 1.54). Signal intensities were normalized to β-actin only, and not to the contralateral hemisphere. All assessments were performed by blinded investigators.

### 2.4. Fluoro-Jade C Staining

Sections were air-dried at 50 °C for 30 min, then immersed in a basic alcohol solution (2 g NaOH + 40 mL H_2_O + 160 mL alcohol) for 5 min. Sections were rinsed in 70% ethanol for 2 min followed by 2 min in water. Brain sections were kept in 0.06% potassium permanganate (Carus Corporation, Peru, IL, USA) for 15 min and rinsed in water for 2 min. Sections were then incubated in working solution of Fluoro-Jade C composed of 2 mL 0.01% Fluoro-Jade C in distilled water and 198 mL 0.1% acetic acid for 10 min, then rinsed in distilled water 3 times. After being dried in a slide warmer, slides quickly dipped into xylene and covered with DPX (Electron Microscopy Sciences, Inc, Hatfield, PA, Cat. No. 13520). Excitation 480 nm and emission 525 nm for green fluorescence. Regions of interest were defined in the basal ganglia at +1.0 mm to +0.5 mm bregma. Three coronal sections per animal were quantified, and positive cells were counted using uniform threshold settings in ImageJ (Version 1.54). All assessments were performed by blinded investigators.

### 2.5. Magnetic Resonance Imaging (MRI) Scanning and T2 Lesion Measurement

Imaging was performed in a 7.0-T Varian MR scanner (183 mm horizontal bore; Varian, Palo Alto, CA, USA). Mice were anesthetized with 2% isoflurane/air mixture throughout the MRI examination. A T2 fast spin-echo (repetition time/echo time = 4000/60 ms, flip angle = 90°, echo train length (ETL) = 8, number of excitations (NEX) = 2, field of view (FOV) = 32 × 32 mm, and inter-slice gap = 0.1 mm) was performed. The field of view was 35 × 35 mm, the matrix was 256 × 128 and in-plane resolution (125 × 250 µm). A total of 25 coronal slices (0.5 mm thick) were acquired to cover the entire cerebrum, from the olfactory bulb to the cerebellum, ensuring full coverage of the ventricles. All image analysis was performed using NIH ImageJ (NIH, Bethesda, MD, USA). T2 lesion displayed high signal intensity on T2 images. Lesion volumes were quantified from T2-weighted images using semi-automated intensity thresholding relative to the contralateral hemisphere (lesion signal ≥ 2 SD above contralateral mean). Motion artifacts were visually inspected and excluded when necessary. Two independent raters, blinded to genotype and time point, performed the segmentation, and inter-rater reliability (ICC = 0.94) is now reported. T2 lesion volume was obtained by combining the high signal intensive area over all slices and multiplying by section thickness (0.5 mm).

### 2.6. Neurological Score

Neurological scores were evaluated at day 1, day 3 and day 7 after SAH using a Garcia’s scoring system as previously described [18]. We evaluated neurological scores using the following six tests as previously described [17]: (1) spontaneous activity; (2) symmetry in the movement of four limbs; (3) forepaw outstretching; (4) climbing; (5) body proprioception; and (6) response to vibrissae touch. Animals were given a score of 3–18 in 1-number steps (if scores are higher, the neurological functions are greater). The same cohort of mice (*n* = 7 per group) was followed longitudinally at days 1, 3, and 7 post-SAH to assess neurological recovery over time, rather than using independent cohorts. Additionally, inter-rater reliability was verified by two blinded investigators scoring a subset of animals independently; the intraclass correlation coefficient (ICC = 0.92) indicated excellent agreement.

### 2.7. Statistical Analysis

In this study, all data are presented as means ± SD. The Shapiro–Wilk test was employed to evaluate the normality of the datasets. The Shapiro–Wilk test has low power when *n* = 3–4. To justify our use of parametric analyses, we visually inspected the residuals and confirmed that data distributions were approximately symmetric, with comparable variances across groups. Data were analyzed by Student’s *t*-test for single comparisons, one-way ANOVA with post hoc Bonferroni-Dunn Correction for multiple comparisons and two-way mixed-effects model (genotype × time) with Holm–Šidák post hoc tests for longitudinal outcomes multiple comparisons. *p* < 0.05 was considered as statistically significant. All statistical analyses were carried out using GraphPad Prism version 8.0 (GraphPad Software Inc., San Diego, CA, USA).

## 3. Results

### 3.1. Lcn2 Expression Was Elevated in Different Parts of Brain at Day 1 After SAH

**Figure 1:**
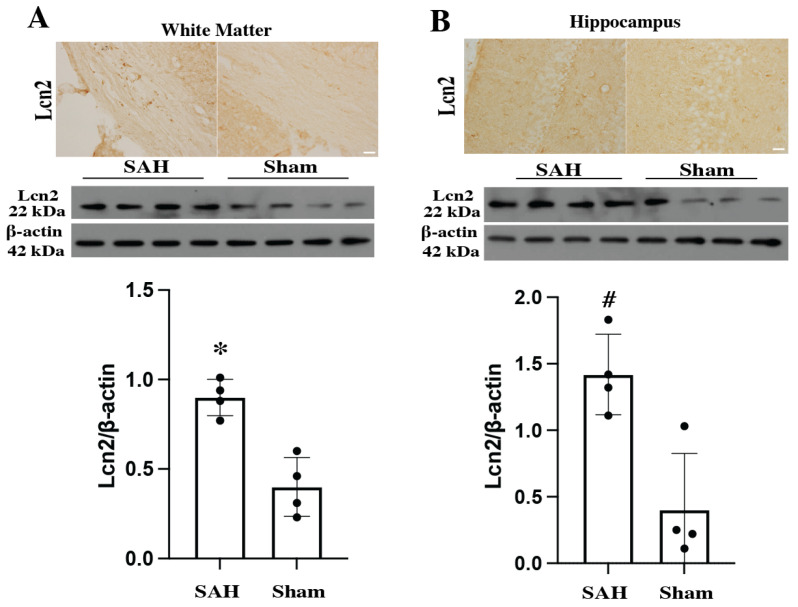
Immunohistochemistry staining and Western blot for Lcn2 expression in different parts of brain compared with sham group at day 1 after SAH in WT animals. (**A**) White matter and (**B**) Hippocampus. (Immunohistochemistry staining: Scale bar = 20 μm) (Western blotting: Values are mean ± SD, *n* = 4 per group, * *p* < 0.05, # *p* < 0.01 by Student’s *t*-test).

Previous studies found white matter [19,20] and hippocampus [21,22] damage after SAH. As demonstrated by several publications, Lcn2 KO mice did not express Lcn2 in the brain [23,24,25]. Immunohistochemistry and Western blotting were used to detect Lcn2 protein expression in the brain after SAH in WT animals (Figure 1). Our immunohistochemistry results showed that Lcn2 expression was upregulated in the white matter and hippocampus on day 1 after SAH, compared to the sham-operated mice. Fewer Lcn2-positive cells were observed in the sham group. Western blot analysis also confirmed that Lcn2 protein levels were significantly higher after SAH compared to the sham group (Uncropped blots are attached in the Supplementary File S1).

### 3.2. Lcn2 Expression Located with Microglia, Astrocytes but Not Neurons

**Figure 2:**
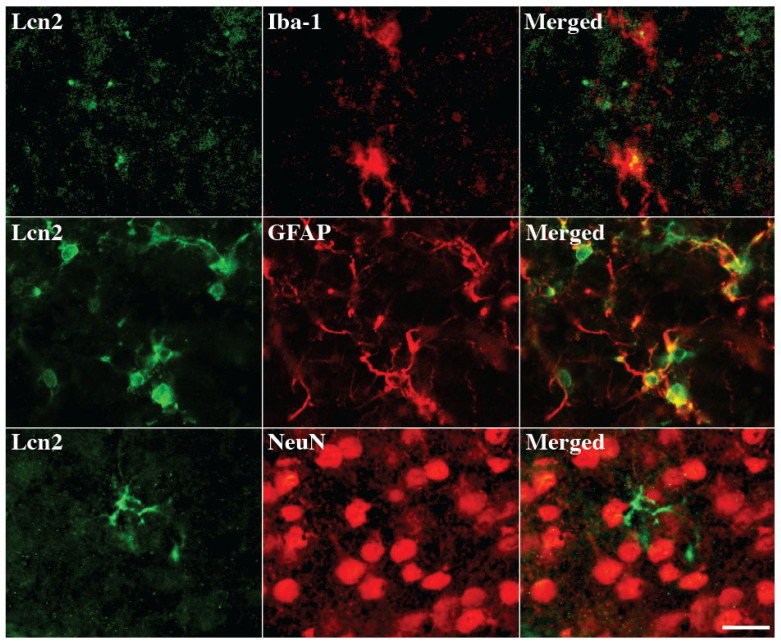
Double-labeling of Lcn2 with Iba-1 (microglia/macrophages marker), GFAP (astrocytes marker), and NeuN (neuronal marker) in the female mouse brain cortex on day 1 after SAH. Scale bar = 20 μm.

Double-labeling was used to determine which cell types express Lcn2 in the cortex after SAH (Figure 2). We found that Lcn2 positive cells predominantly colocalized with GFAP positive astrocytes after SAH. Less Lcn2 positive cells colocalized with Iba-1 positive microglia/macrophages. No Lcn2 positive cells colocalized with NeuN positive neurons. These results suggested Lcn2 is mainly expressed in astrocytes after SAH, which was consistent with our previous findings in a rat ICH model [26].

### 3.3. Lcn2 KO Reduced Heme Oxygenase-1 and Ferritin Upregulation at Day 1 After SAH

**Figure 3:**
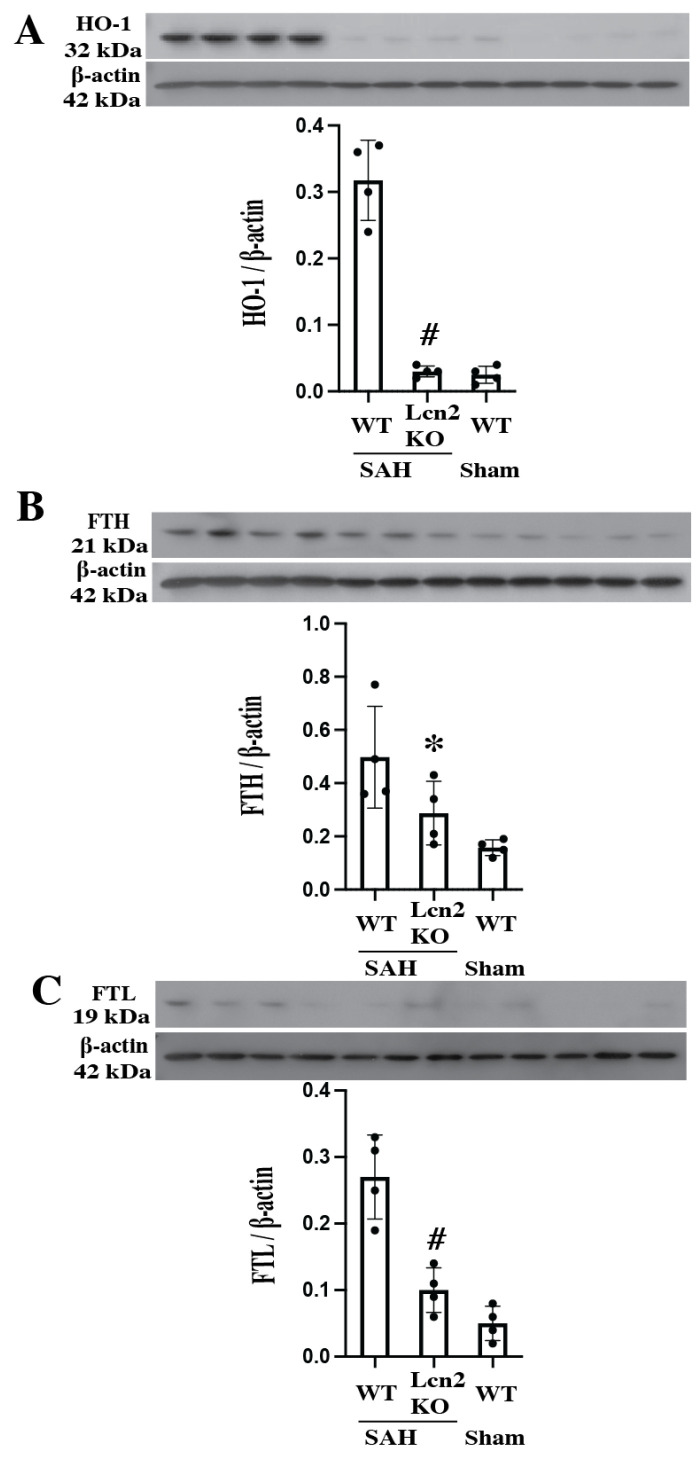
HO-1 (**A**), FTH (**B**) and FTL (**C**) protein levels in the right cerebral hemisphere of WT and Lcn2 KO mice 24 h after SAH or sham operation. Values are mean ± SD, *n* = 4 per group, # *p* < 0.01 versus WT SAH group, * *p* < 0.05 versus WT SAH group by one-way ANOVA with post hoc Bonferroni-Dunn Correction.

The SAH severity scores were not significantly different between WT and Lcn2 knockout animals (9 ± 2 in WT mice and 8 ± 3 in Lcn2 KO mice; *p* > 0.05). Heme oxygenase (HO)-1 plays an important role after SAH degrading heme into iron, biliverdin, and carbon monoxide. It is markedly upregulated after SAH in WT mice (Figure 3A). In Lcn2 KO mice, HO-1 expression was reduced ~11-fold compared with WT mice (HO-1/β-actin: 0.03 ± 0.01 vs. 0.34 ± 0.03; *p* < 0.01).

The iron release from heme degradation can be bound by endogenous iron chelators. Our previous study [27] demonstrated that endogenous ferritin plays a vital role in iron chelation after ICH, with both H- and L-chain protein levels progressively increasing over time. In the present study, ferritin upregulation was significantly attenuated in Lcn2 KO mice, as shown by Western blot analysis. Ferritin H-chain expression was reduced in Lcn2 KO mice compared with WT mice (FTH/β-actin: 0.29 ± 0.12 vs. 0.50 ± 0.14; *p* < 0.05) (Figure 3B), as was ferritin L-chain expression (FTL/β-actin: 0.10 ± 0.04 vs. 0.27 ± 0.08; *p* < 0.01) (Figure 3C). Together, these results suggest that loss of Lcn-2 reduces iron overload after SAH.

### 3.4. SAH-Induced Smaller MRI T2 Lesion, Less BBB Disruption and Decreased Neuronal Death in Lcn2 KO Mice

**Figure 4:**
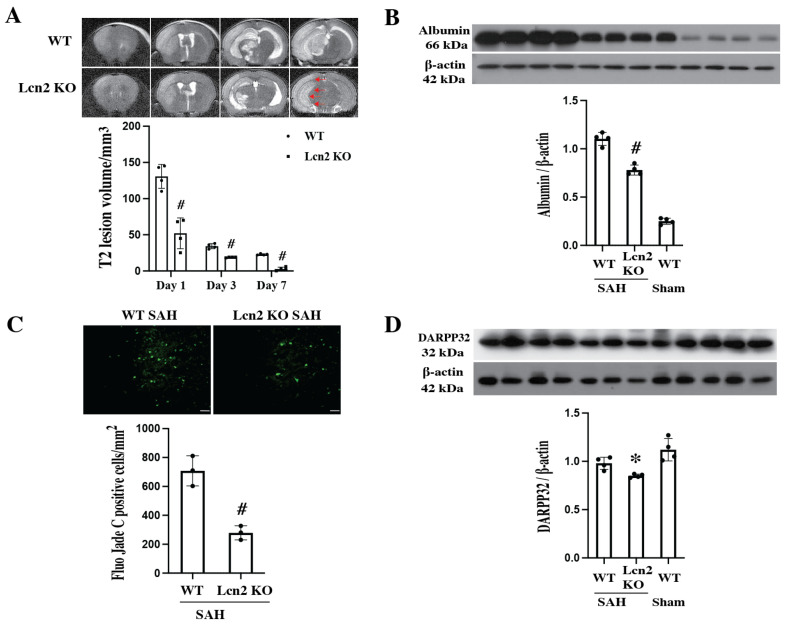
(**A**) Representative MRI T2 lesion sections in WT and Lcn2 KO mice at day 1 after SAH. Lesion volumes were quantified at days 1, 3, and 7. Data are presented as mean ± SD (*n* = 4 per group). # *p* < 0.01 vs. WT SAH group by two-way mixed-effects model (genotype × time) with Holm–Šidák post hoc test (interaction *p* < 0.0001, 95% CI (25.27–50.89)). Red arrows indicate T2 lesion. (**B**) Albumin protein levels in the right cerebral hemisphere of WT and Lcn2 KO mice 24 h after SAH or sham operation. Data are mean ± SD (*n* = 4 per group). # *p* < 0.01 vs. WT SAH group by one-way ANOVA with post hoc Bonferroni-Dunn Correction. (**C**) Quantification of Fluoro-Jade C–positive cells in the right basal ganglia 24 h after SAH in WT and Lcn2 KO mice. Data are mean ± SD (*n* = 3 per group). # *p* < 0.01 vs. WT SAH group by Student’s *t*-test. Scale bar = 20 μm. (**D**) Protein levels of DARPP-32 in the right basal ganglia 24 h after SAH. Data are mean ± SD (*n* = 4 per group). * *p* < 0.05 vs. Lcn2 KO and WT sham groups by one-way ANOVA with post hoc Bonferroni-Dunn Correction.

SAH-induced MRI T2 lesions, BBB disruption, and neuronal death (Figure 4). T2 lesion volumes decreased from day 1 to day 7 in both WT and Lcn2 KO mice. However, lesion sizes were consistently smaller in Lcn2 KO mice compared with WT mice (Day 1: 52.1 ± 23.9 mm^3^ vs. 130.7 ± 19.4 mm^3^; Day 3: 18.7 ± 0.6 mm^3^ vs. 34.3 ± 4.0 mm^3^; Day 7: 2.7 ± 4.0 mm^3^ vs. 22.9 ± 1.1 mm^3^; # *p* < 0.01).

BBB disruption was assessed by albumin Western blot analysis. SAH-induced significant albumin leakage quantified by calibrated densitometry (normalized to housekeeping protein β-actin) in WT mice, which was markedly reduced in Lcn2 KO mice (albumin/β-actin: 0.78 ± 0.03 vs. 1.11 ± 0.06 in WT mice; *p* < 0.01). Albumin was barely detectable in the sham group.

Neuronal death was evaluated using Fluoro-Jade C staining, which labels cell bodies, dendrites, and axons of degenerating neurons. Fluoro-Jade C–positive cells were observed in the basal ganglia on day 1 after SAH. The number of positive cells was significantly reduced in Lcn2 KO mice compared with WT mice (279 ± 63 cells/mm^2^ vs. 708 ± 109 cells/mm^2^; *p* < 0.01).

DARPP-32, a marker expressed in neuronal cell bodies and dendrites, and a major downstream target of dopamine-activated adenylyl cyclase in the striatum, was also examined. Our previous study [28] established DARPP-32 as a sensitive marker of neuronal injury in the basal ganglia following ICH. In the present study, Western blot analysis demonstrated that Lcn2 KO preserved more neuronal survival after SAH (DARPP-32/β-actin: 0.98 ± 0.07 vs. 0.85 ± 0.02 in WT mice; *p* < 0.05).

### 3.5. SAH Resulted in Reduced Neurological Deficits in Lcn2 KO Mice

**Figure 5:**
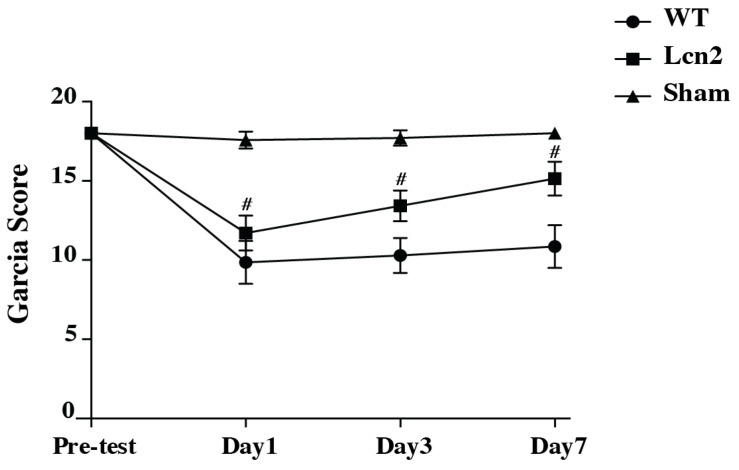
Garcia scores assessing neurological deficits in WT and Lcn2 KO mice after SAH, and in WT sham mice before surgery and at days 1, 3, and 7. Values are mean ± SD, *n* = 7 per group, # *p* < 0.01, compared with WT SAH group by two-way mixed-effects model (genotype × time) with Holm–Šidák post hoc test (interaction *p* < 0.0001, 95% CI (−2.823–1.748)).

Both WT and Lcn2 KO mice exhibited significant neurological deficits at day 1 after SAH, as assessed by the modified Garcia scoring system. However, Lcn2 KO mice demonstrated significantly fewer deficits compared with WT mice at day 1 (11 ± 1 vs. 9 ± 1, # *p* < 0.01; Figure 5), day 3 (13 ± 1 vs. 10 ± 1, # *p* < 0.01; Figure 5) and day 7 (15 ± 1 vs. 11 ± 1, # *p* < 0.01; Figure 5).

## 4. Discussion

These are the following findings in this study on female mice: (1) Lcn2 expression was significantly upregulated in white matter and hippocampus after SAH. (2) Lcn2 was primarily localized to astrocytes, with limited expression in microglia and no detectable expression in neurons. (3) Lcn2 KO mice exhibited reduced oxidative stress, smaller MRI T2 lesions, decreased BBB disruption, attenuated neuronal death, and improved neurological function compared with WT mice. Collectively, these results suggest that Lcn2 contributes to early brain injury after SAH in female mice. In combination with results from male mice, these results indicate that Lcn2 may represent an important therapeutic target for reducing secondary brain injury.

Lcn2 is a secretory protein that regulates diverse cellular processes. One clinical study found that CSF Lcn2 levels increased early after SAH and were significantly higher than in non-SAH controls [15]. Elevated CSF Lcn2 correlated with inflammatory markers and was independently associated with worse functional outcomes at 3 and 6 months. Our findings are consistent with previous work showing that Lcn2 is strongly induced by brain injury and inflammation. Earlier studies demonstrated that Lcn2 expression increases after ICH, where it contributes to neuronal injury and White matter damage [13]. Our previous research [19,20] has verified Lcn2 expression was significantly increased in white matter after SAH in WT male mice and Lcn2 deficiency attenuated BBB disruption, myelin and axonal damage and oligodendrocyte loss, demonstrating Lcn2 may have potential role on BBB disruption in white matter after SAH. The present study extends these observations to SAH, revealing that Lcn2 is also robustly upregulated in this condition and is associated with brain injury severity in female mice. Lcn2 can be secreted by astrocytes under inflammatory conditions [29], and in the injured hippocampus, Lcn2 is up-regulated in the reactive astrocytes after kainite administration [30]. In spinal cord injury, high Lcn2 expression was induced in astrocytes but not in microglia [31]. The high expression of Lcn2 in astrocytes after SAH aligns with prior reports that astrocytes are a major source of Lcn2 in the central nervous system under pathological conditions. Astrocyte-derived Lcn2 may act as a signaling molecule, amplifying inflammatory cascades, associated with promoting iron dysregulation, and exacerbating oxidative stress, thereby contributing to neuronal damage. Future studies employing cell type-specific conditional knockout models (e.g., Aldh1l1-CreER or Gfap-Cre-mediated deletion of Lcn2), or pharmacological strategies such as 24p3R receptor blockade or neutralizing antibodies against Lcn2, will be essential to confirm whether astrocyte-derived Lcn2 directly drives post-SAH iron dysregulation and neurotoxicity. Lcn2 plays a critical role in iron homeostasis by binding iron-loaded siderophores and transporting iron into cells via the 24p3R receptor, thereby contributing to intracellular iron accumulation and oxidative stress [32]. In the injured brain, astrocyte-derived Lcn2 acts as a proinflammatory mediator that amplifies cytokine production and glial activation [33]. Furthermore, Lcn2 has been shown to enhance reactive oxygen species production and mitochondrial dysfunction in neural cells under stress conditions [34]. Estrogen has been shown to modulate both Lcn2 transcription and secretion through estrogen receptor-mediated signaling, influencing iron metabolism, oxidative stress responses, and glial activation [35,36,37]. Previous studies report that 17β-estradiol show neuroprotection after injury [38]. This hormonal regulation may contribute to the sex-specific neuroprotection observed in females following SAH.

Oxidative stress is a key mediator of early brain injury following SAH [39]. Heme degradation products released after hemorrhage are metabolized by heme oxygenase-1 (HO-1) into biliverdin, iron, and carbon monoxide, leading to free iron accumulation and generation of reactive oxygen species [40]. The reactivation of HO-1 is part of the acute-phase response to brain injury. In this study, HO-1 expression was markedly reduced in Lcn2 KO mice, suggesting that Lcn2 deficiency dampens heme degradation-related oxidative stress. Ferritin, a ubiquitous intracellular protein composed of 24 subunits of heavy (FTH) and light (FTL) chains, normally serves to store iron and release it in a controlled fashion as a compensatory mechanism to sequester excess iron. Ferritin upregulation was also attenuated in Lcn2 KO mice, indicating that Lcn2 contributes to post-SAH iron overload and oxidative stress. Lcn2 regulates iron uptake by binding iron-loaded siderophores and transferring them into cells through its receptor 24p3R, leading to intracellular iron accumulation [32]. Gene expression of both Lcn2 and ferritin subunits is upregulated by iron, further amplifying iron-mediated toxicity [41]. Excessive intracellular iron accumulation contributes to oxidative damage and neuronal injury. Thus, blocking Lcn2-dependent iron trafficking and ferritin synthesis may represent an effective strategy to limit oxidative stress and reduce brain injury after SAH.

Disruption of the BBB is another hallmark of early brain injury after SAH and contributes to vasogenic edema, neuroinflammation, and poor neurological outcomes [39]. In this study, BBB disruption was significantly reduced in Lcn2 KO mice, as evidenced by decreased albumin leakage. This suggests that Lcn2 exacerbates BBB injury, potentially through astrocyte-mediated inflammatory signaling and iron-driven oxidative stress. Maintenance of BBB integrity in Lcn2 KO mice likely contributed to the smaller T2 lesions and probably reduced edema observed on MRI, further supporting the protective effect of Lcn2 deficiency.

Neuronal death is a major determinant of neurological dysfunction after SAH [8]. Our Fluoro-Jade C staining results revealed significantly fewer degenerating neurons in Lcn2 KO mice compared with WT mice, indicating that Lcn2 deletion conferred neuroprotection. In addition, DARPP-32, a sensitive marker of neuronal integrity in the basal ganglia [28], was relatively preserved in Lcn2 KO mice. These results highlight the importance of Lcn2 in mediating neuronal loss after SAH and suggest that targeting Lcn2 may protect against neurodegeneration in vulnerable brain regions such as the basal ganglia. Neurological outcomes, as measured by Garcia’s scoring system, further confirmed the detrimental role of Lcn2 in SAH. Although both WT and KO mice exhibited deficits at day 1, Lcn2 KO mice demonstrated significantly better recovery by days 3 and 7. This behavioral improvement is consistent with the observed reductions in oxidative stress, BBB disruption, and neuronal death, and it underscores the functional relevance of Lcn2-mediated injury pathways.

Pharmacological targeting of Lcn2 has emerged as a potential therapeutic strategy in neurological disorders [42,43]. Given its role in mediating iron trafficking, oxidative stress, neuroinflammation, and BBB disruption, Lcn2 represents an attractive target for intervention. Approaches such as neutralizing antibodies against Lcn2, inhibition of its receptor 24p3R, or small molecules that block Lcn2–iron interactions could attenuate downstream injury pathways. Although most evidence to date comes from genetic knockout models, translation of these findings into pharmacological therapies may provide a novel avenue to reduce early brain injury and improve outcomes after SAH. Given that Lcn2 regulates iron metabolism, innate immunity, and neutrophil function, systemic inhibition may disrupt iron homeostasis or increase infection risk, highlighting the need to determine an optimal post-SAH treatment window that balances early neuroprotection with preservation of iron clearance and tissue repair.

Several limitations of this study should be acknowledged. First, while we demonstrated that female Lcn2 KO mice are protected against early brain injury after SAH, the precise molecular mechanisms by which Lcn2 exacerbates injury remain incompletely defined. Future studies should examine downstream signaling pathways. Second, our experiments focused on early outcomes (up to 7 days after SAH). Longer-term studies are needed to assess the role of Lcn2 in chronic brain injury and functional recovery. Third, some experiments were performed with relatively small group sizes (*n* = 3–4), which may limit statistical power. This limitation primarily reflects the exploratory nature of the study and our effort to adhere to institutional and national guidelines for minimizing animal use in accordance with the principles of the 3Rs (Replacement, Reduction, and Refinement). Nevertheless, the sample sizes used are consistent with prior studies employing mouse models of SAH [19,20], and the results demonstrated consistent trends across independent experiments and multiple complementary assays. Fourth, exclusive use of a genetic KO model represents a limitation regarding translational applicability. While the Lcn2 knockout model provides a precise approach to delineate the biological role of Lcn2 in early brain injury after SAH, pharmacological inhibition of Lcn2 or its receptor (24p3R) would indeed enhance clinical relevance. Previous studies have shown that neutralizing antibodies or small-molecule inhibitors targeting Lcn2 signaling can effectively reduce neuroinflammation and oxidative stress in other neurological conditions [44,45]. Future studies using pharmacological blockade of Lcn2 or 24p3R are warranted to validate its therapeutic potential in SAH. Finally, while knockout models provide valuable insights, pharmacological inhibition of Lcn2 or its receptor would strengthen the translational potential of these findings.

In conclusion, this study demonstrates that in female mice, Lcn2 is upregulated in the brain after SAH, primarily in astrocytes, and contributes to oxidative stress, BBB disruption, neuronal death, and neurological deficits. Lcn2 deficiency mitigates these pathological processes and improves functional recovery, highlighting Lcn2 as a promising therapeutic target for early brain injury after SAH. Targeting Lcn2-mediated pathways, particularly those involving iron dysregulation and astrocytic signaling, may offer new strategies for improving outcomes in patients suffering from SAH. With the greater prevalence of SAH in women, showing that Lcn2 deletion is protective in female animals is an important step for translation.

## Data Availability

Uncropped blots are provided in Supplementary File S1; additional data is available upon request.

## Supplementary Materials

The following supporting information can be downloaded at: https://www.mdpi.com/article/10.3390/cells14221770/s1, File S1: uncropped blots.

## Abbreviations

The following abbreviations are used in this manuscript:

SAH	Subarachnoid hemorrhage
BBB	Blood–brain barrier
Lcn2	Lipocalin 2
NGAL	Neutrophil gelatinase-associated lipocalin
FTL	Ferritin light chain
FTH	Ferritin heavy chain
KO	Knockout
WT	Wild type
ICH	Intracerebral hemorrhage
CSF	Cerebrospinal fluid
MRI	Magnetic resonance imaging
SD	Standard deviation
ECA	External carotid artery
CCA	Common carotid artery
ICA	Internal carotid artery
HO-1	Heme oxygenase-1
