# Supplementary material for: Role of Lipocalin-2 in Brain Injury After Subarachnoid Hemorrhage in Female Mice
Source: Cells. 2025 Nov 12;14(22):1770. doi: 10.3390/cells14221770 (PMC12651340; doi:10.3390/cells14221770)
Supplement: Supplementary file 1 [file cells-14-01770-s001.zip › Uncropped blots/Figure 3/Fgiure 3 FTH band.pdf]

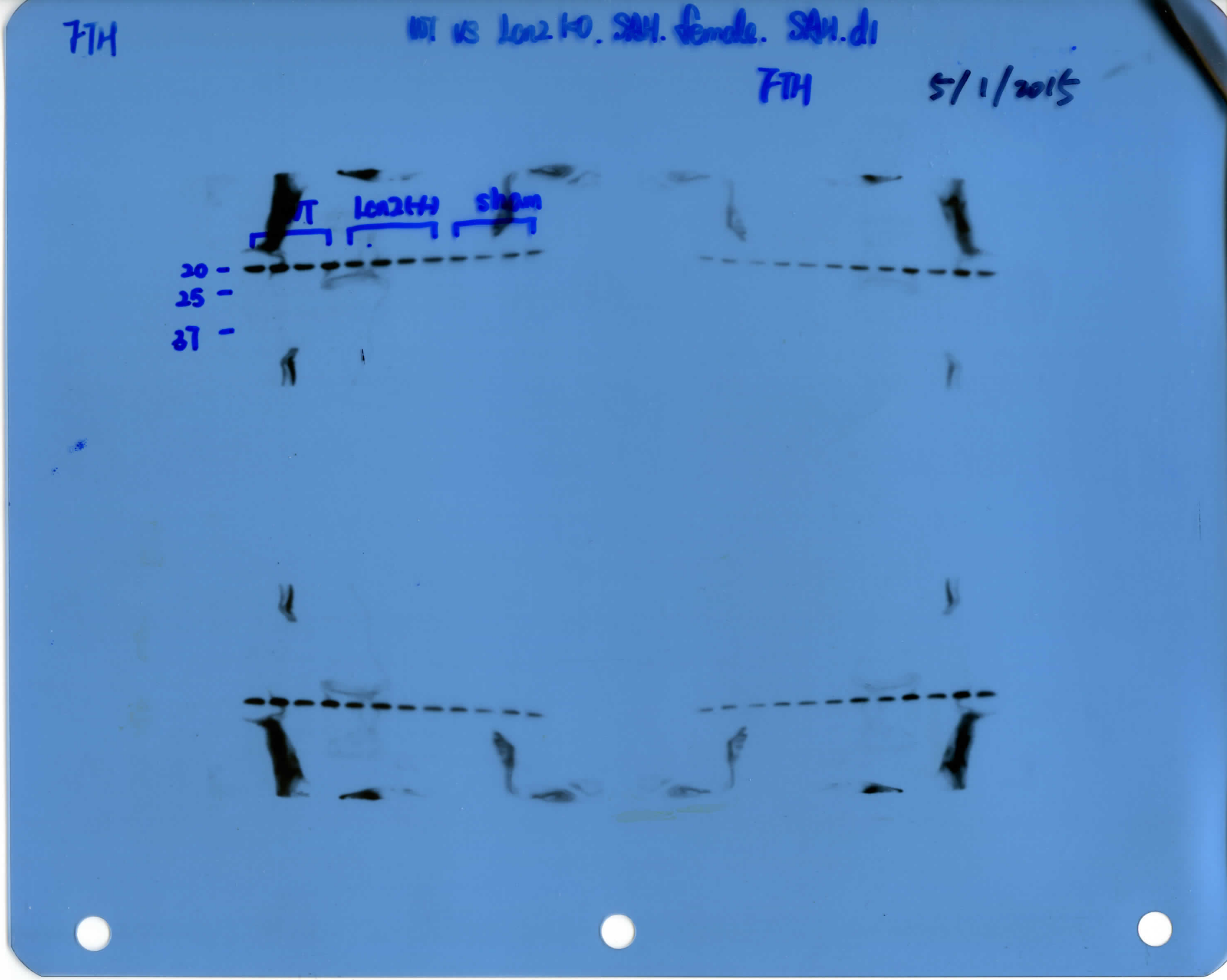
FTH
WT vs Lcn2 KO. SAH. female. SAH.d1
FTH
5/1/2015
WT
Lcn2-/-
sham
20 -
25 -
37 -
