# Supplementary material for: Role of Lipocalin-2 in Brain Injury After Subarachnoid Hemorrhage in Female Mice
Source: Cells. 2025 Nov 12;14(22):1770. doi: 10.3390/cells14221770 (PMC12651340; doi:10.3390/cells14221770)
Supplement: Supplementary file 1 [file cells-14-01770-s001.zip › Uncropped blots/Figure 3/Figure 3 FTL and FTH corresponding B-actin bands.pdf]

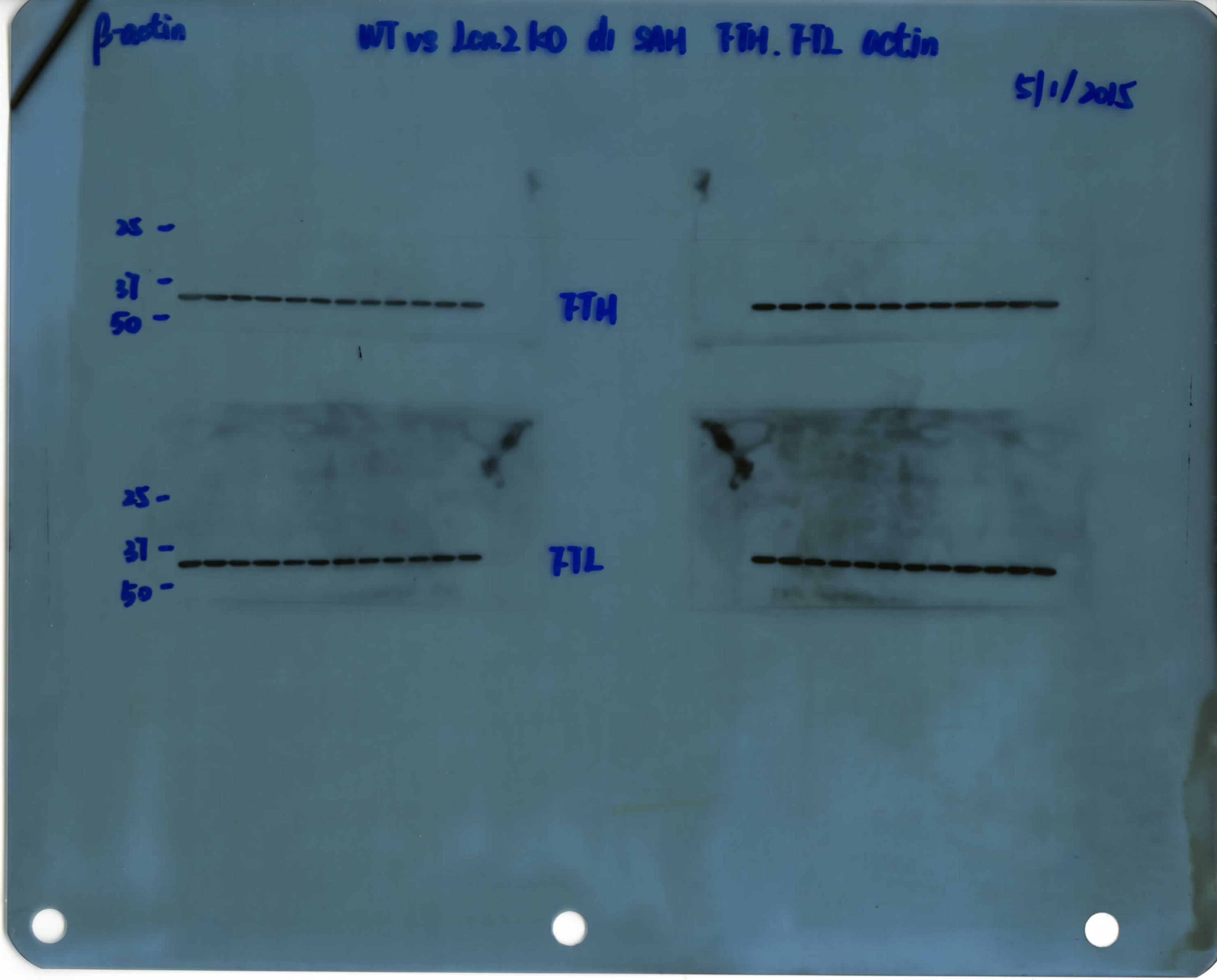
β-actin
WT vs Lcn2 KO di SAH FTH. FTL actin
5/1/2015
25 -
37 -
50 -
FTH
25 -
37 -
50 -
FTL
