# Supplementary material for: Role of Lipocalin-2 in Brain Injury After Subarachnoid Hemorrhage in Female Mice
Source: Cells. 2025 Nov 12;14(22):1770. doi: 10.3390/cells14221770 (PMC12651340; doi:10.3390/cells14221770)

WT. vs. Lcn2 KO SAM. female d1.

5/6/2015

β-actin

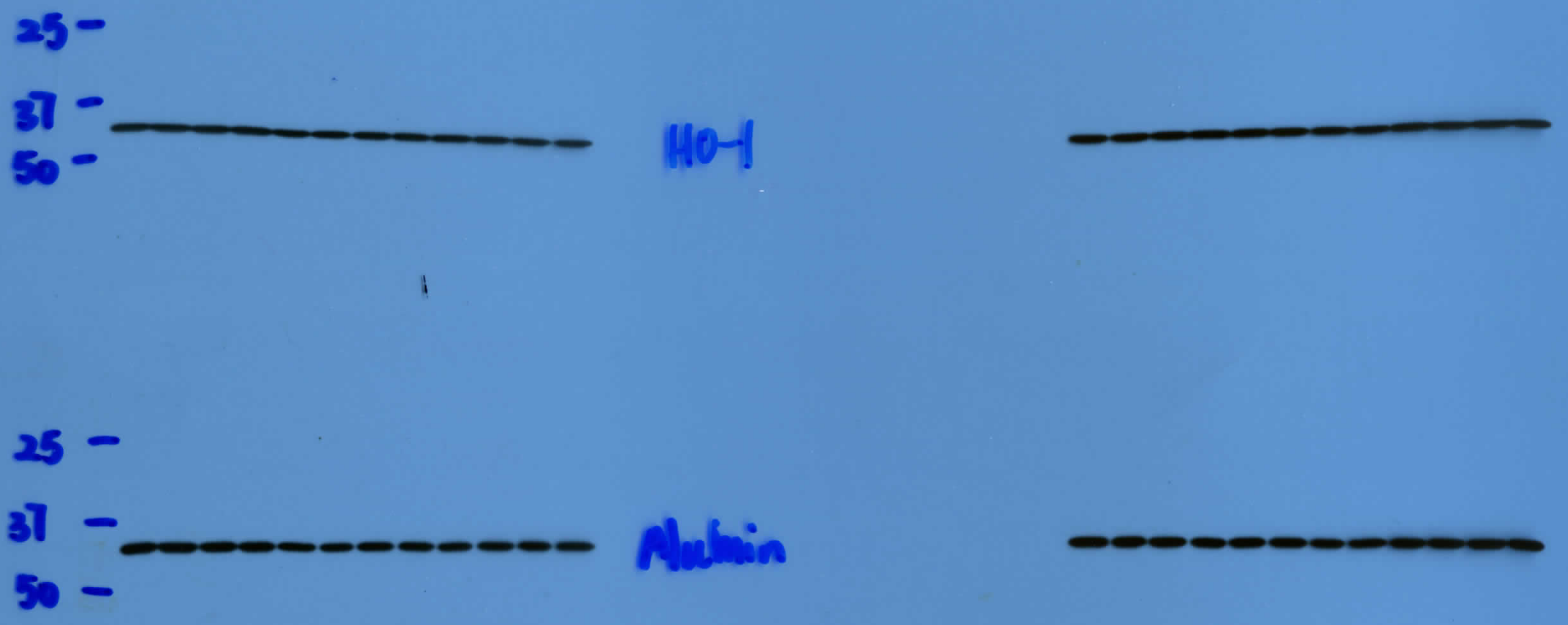

Supplement: Supplementary file 1 [file cells-14-01770-s001.zip › Uncropped blots/Figure 3/Figure 3 HO-1 corresponding B-actin band.pdf]
