# Supplementary figures and images for: Role of Lipocalin-2 in Brain Injury After Subarachnoid Hemorrhage in Female Mice
Source: Cells. 2025 Nov 12;14(22):1770. doi: 10.3390/cells14221770 (PMC12651340; doi:10.3390/cells14221770)

dl Lcn2  $\beta$ -actin 4/28/15

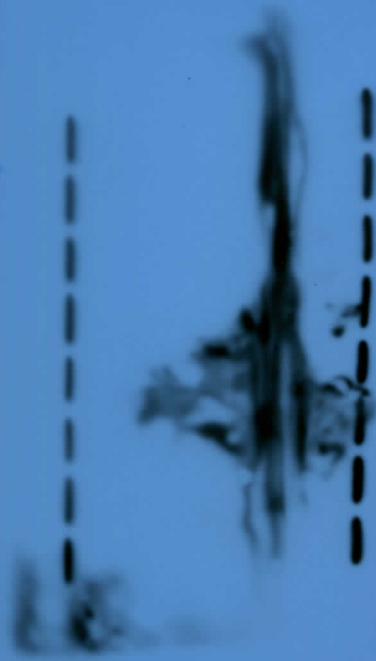

SAH  
W 25 -  
37 -  
50 -  
WT sham

37 -  
50 -

sham WT

H 25 -  
37 -  
50 -

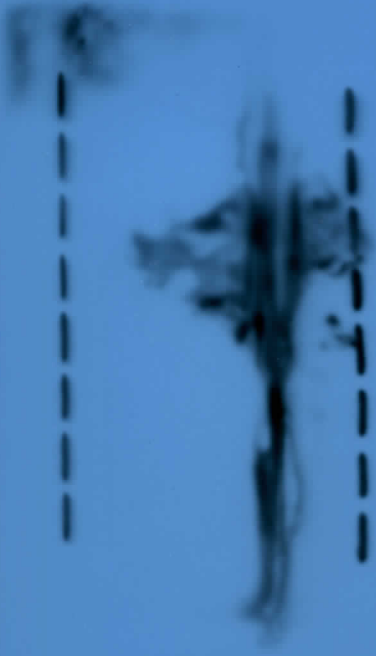

37 -  
50 -

37 -  
50 -

Supplement: Supplementary file 1 [file cells-14-01770-s001.zip › Uncropped blots/Figure 1/Figure 1 LCN2 corresponding B-actin bands.pdf]

H0-1

SNH. WT vs  $lca2(-/-)$  female. H0-1

5/02/17/5

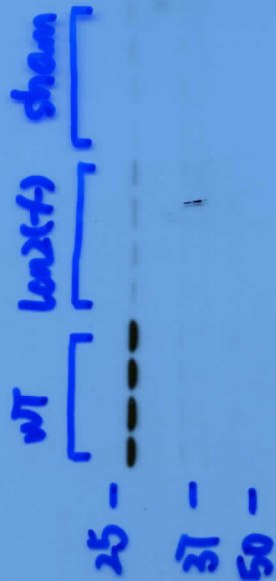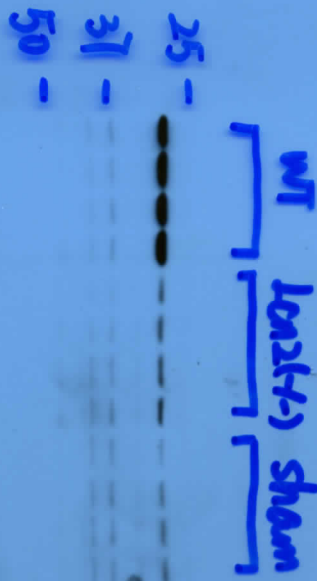

Supplement: Supplementary file 1 [file cells-14-01770-s001.zip › Uncropped blots/Figure 3/Figure 3 HO-1 band.pdf]

Albumin

WT vs Lcn2(-/-) stain di

5/13/2015

WT Lcn2(-/-) sham

37

50

75

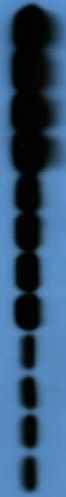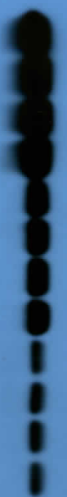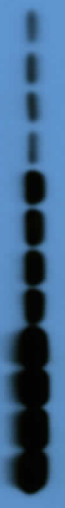

Supplement: Supplementary file 1 [file cells-14-01770-s001.zip › Uncropped blots/Figure 4/Figure 4 Albumin band.pdf]

dl OAGP-32

7/16/2015

1b

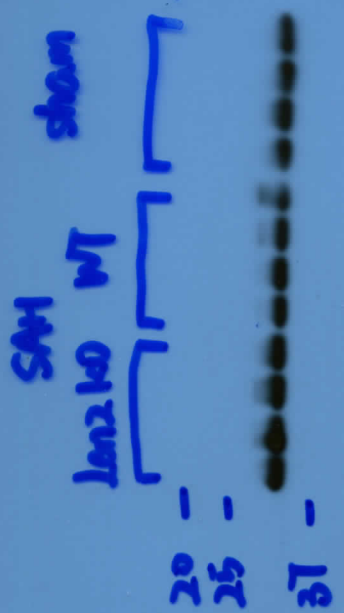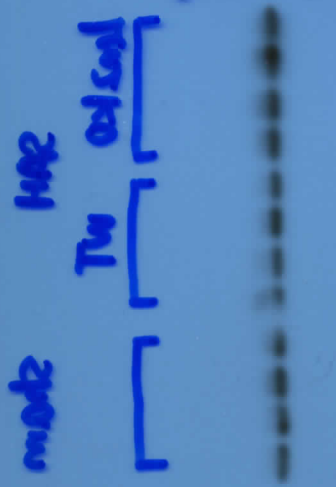

✓

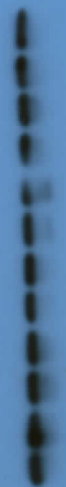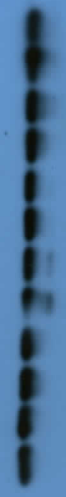

Supplement: Supplementary file 1 [file cells-14-01770-s001.zip › Uncropped blots/Figure 4/Figure 4 DARPP32 band.pdf]
